# Association of workplace support for health with occupational health literacy and illness avoidance: moderated mediation by functioning through a salutogenic lens

**DOI:** 10.1186/s12889-025-21831-3

**Published:** 2025-08-16

**Authors:** Nestor Asiamah, Isaac Aidoo, Etornam Doamekpor, Emelia Sarpong, Emelia Danquah, Cosmos Yarfi, Eric Eku, Usman Yaw Baidoo, Christiana Afriyie Manu, Rita Sarkodie Baffoe

**Affiliations:** 1 Division of Interdisciplinary Research and Practice, School of Health and Social Care, University of Essex, Colchester, Essex CO4 3SQ UK; 2 International Public Health Management Programme, University of Europe for Applied Sciences, Reiterweg 26B, 58636 Iserlohn, Germany; 3 Research Faculty, Berlin School of Business and Innovation, 97-99 Karl Marx Strasse, 12043 Berlin, Germany; 4 Department of Geriatrics and Gerontology, Africa Center for Epidemiology, P. O. Box AN 18462, Accra, Ghana; 5 Department of Building Technology, Accra Technical University, P.O. Box GP 561, Accra, Ghana; 6 Faculty of Business Administration, KAAF University College, Central Region, P.O. Box Wu 177, Fetteh Kakraba, Ghana; 7 School of Business, Accra Technical University, Barnes Road, Accra Metro, P. O Box GP 561, Accra, Ghana; 8 Koforidua Technical University, Koforidua, Ghana; 9 Department of Physiotherapy and Rehabilitation Sciences, University of Health and Allied Sciences, PMB 31, Ho, Ghana; 10 Department of Secretaryship and Management Studies, Dr Hilla Limann Technical University, Upper West, Wa, Ghana; 11 Department of Business and Social Sciences Education, University of Cape Coast, Private Mail Bag, Cape Coast, Ghana

**Keywords:** Workplace support for health, Functioning, Illness avoidance, Occupational health literacy, Sex, Older adults

## Abstract

**Background:**

An increase in the proportion of older employees over the coming decades is an outcome of ageing of the world’s population. Workplace interventions that enable older employees to maintain work productivity and avoid illness are, therefore, increasingly important. An aspect of these interventions is Workplace Support for Health (WSH), which fosters Occupational Health Literacy (OHL) and encourages health behaviours in an organization. Common health behaviours are healthy diet and physical activity, both of which protect physical functioning and well-being. Employees are more likely to avoid illness and maintain physical functioning if they receive enough WSH and improve their OHL.

**Aim:**

This study aimed to investigate whether there is a moderated mediation by functioning in the relationship between WSH, OHL, and illness avoidance.

**Methods:**

A cross-sectional design with sensitivity analyses and measures against common methods bias was adopted. The participants were 1015 middle-aged and older adult employees aged 50 to 85 years. The participants were workers of public and private organizations in Accra, Ghana. The main variables (i.e., WSH, OHL, functioning, and illness avoidance) were measured with Likert-type scales adopted in whole from the literature. Data were analysed with Hayes’ Process Model through structural equation modelling.

**Results:**

WSH had a positive effect on functioning (β = 0.29; *p* < 0.001) and illness avoidance (β = 0.25; *p* < 0.001) in the whole sample. Functioning had a positive effect on illness avoidance (β = 0.45; *p* < 0.001). A positive indirect effect of WSH (through functioning) on illness avoidance was confirmed. Evidence of a moderated mediation was found, suggesting that the indirect effect of WSH on illness avoidance was stronger at higher OHL. Our sensitivity analysis yielded similar effects in men and women.

**Conclusion:**

WSH can enable older employees to improve their physical functioning and avoid illness, especially if it fosters higher OHL. WSH can be an appropriate way to protect employee health in response to ageing of the workforce.

**Supplementary Information:**

The online version contains supplementary material available at 10.1186/s12889-025-21831-3.

## Introduction

The ageing of the world’s population accompanies opportunities and challenges. A noteworthy challenge is an increase in the burden of non-communicable diseases and their national healthcare cost [1, 2]. On the flip side, ageing allows organizations to retain their workforce for a longer period if employees maintain optimal health over the life course. Given that life expectancy is increasing globally due to population ageing [3], workplace programmes enabling employees to avoid illness are essential. Employees can maintain productivity and avoid early retirement in organizations where these programmes support healthy longevity. Programmes aimed at supporting employees to maintain health are encapsulated in the term “workplace health promotion” [4, 5].

Workplace health promotion aims to support employees to avoid illness or disease [4, 6] and encompasses pro-health interventions such as workplace design to encourage Physical Activity (PA). An aspect of workplace health promotion is Workplace Support for Health (WSH), which is the availability of individuals, policies, and resources within a workplace that encourage employees to value their health, perform health-seeking behaviours, and avoid illness [7, 8]. WSH has been upheld in the literature as a productivity-driven organizational practice [8, 9], which has encouraged the development of a psychometric tool measuring it [9]. An admirable attribute of WSH is that it advocates a salutogenic pathway to healthy workplaces [10], which means it focuses on the determinants of health rather than the causes of disease. Suffice it to say that WSH aims to enable employees to avoid illness by maintaining health-seeking behaviours. *Illness avoidance* is the individual’s maintenance of health in the absence of illness and without dependence on prescribed medication [11].

Research to date has shown that workplace health promotion encourages health-seeking behaviours such as health-related social activity participation, PA, and a healthy diet [4, 12, 13]. Empirical evidence also exists on the positive effect of workplace health promotion on health. A cross-sectional study in the USA, for example, reported a positive association between WSH and well-being proxied with job satisfaction and the absence of burnout [5]. WSH was shown to predict vitality, health, and a reduction in absences from work due to ill health [13]. Interventions providing WSH have also been confirmed to improve well-being and health-seeking behaviours [12, 13], but no study has explored the potential effect of WSH on physical functioning and illness avoidance. This gap is worth filling because illness avoidance is the basic goal of workplace health promotion, and people who sufficiently perform health-seeking behaviours (e.g., PA) and report good health are not necessarily without illnesses. Functioning is necessary for work engagement and productivity; hence, its maintenance across the lifespan is necessary.

WSH includes activities that can enhance Occupational Health Literacy (OHL), which is the degree to which employees can obtain, communicate, process, and understand information regarding health and safety as well as services in making good health-related decisions at work [14]. One’s well-being is influenced by their ability to make good decisions about health, so OHL can enable individuals to maintain health. Research has confirmed that workplace health promotion efforts are associated with higher OHL [14, 15], which means OHL can be higher among employees with higher WSH. Because it encourages health-seeking behaviours such as PA and healthy eating [12], WSH can have a positive effect on functioning. Research suggests that functioning is necessary for avoiding disease [16, 17]; hence, it can have a positive effect on illness avoidance. We reason from these perspectives that OHL can interact with WSH to positively influence illness avoidance, and there is a potential moderation of the indirect effect of WSH on illness avoidance (through functioning) by OHL. This effect of OHL is a *moderated mediation* [18] that needs to be evaluated, given the above research gap.

This study, therefore, assessed the above moderated mediation using the Salutogenic Model [19, 20]. By our use of a salutogenic approach, we provided evidence for future research by which organizations can support ageing employees to avoid illness since efforts for preventing disease are superior to programmes focused on the cure or treatment of disease [21, 22]. This study was the first to test a nexus involving WSH and illness avoidance. Our analysis was strengthened with two sensitivity analyses, including an analysis of the consistency of the evidence between men and women. Owing to differences in ageing, workplace opportunities, and health indicators for men and women [23, 24], fitting the moderated mediation model between these groups is a good way to ascertain the stability of the effects. Previous studies [25, 26] followed a similar technique to investigate the consistency of effects. By focusing on older employees, this study aimed to elucidate implications for workplace health promotion in response to the ageing of the workforce.

## Theoretical framework

Measures of WSH embody items measuring the availability of health champions, support for healthy-seeking behaviours, and policies that support employee health [9]. These items reflect a salutogenic process of WSH that emphasizes illness prevention among employees. This attribute of WSH motivated us to adopt the Theory of Salutogenesis (also referred to as the Salutogenic Model) as a lens for developing our moderated mediation model. This model was propounded by medical sociologist Aaron Antonovsky to describe the process by which organizations can promote health by focusing on factors that maintain or enhance well-being rather than on factors that cause disease [19, 27].

As a positive theory of health, the model posits that life experiences and people’s view of themselves influence their health. The model delineates practices that promote the effective use of individual and collective resources to improve well-being. These practices originate from life experiences that enable the individual to act and make decisions to avoid disease. For example, an individual may make use of resources to maintain a trajectory of PA to avoid the onset of chronic diseases. Resources include social networks that encourage PA, money for paying gymnasium fees, and the physical strength needed to exercise. The salutogenic model overlaps with the Continuity Theory of Ageing (CTA) developed by Robert Atchley [28] as well as the Activity Theory of Ageing (ATA) proposed by Robert Havighurst [29] with its recognition of life experiences as enablers of pro-health action, decisions, and choices. The ATA and CTA posit that the maintenance of social and physical activities across the lifespan is implicit in life experiences and the ability to adapt to current challenges with these experiences.

One of the attributes of the salutogenic model that has been criticized is its complexity, which has made its application in practice difficult [19, 30]. As a result, many researchers have adapted this model into practice-oriented paradigms for promoting health [31, 32]. A facet of the model frequently adopted is its concept of Sense of Coherence (SOC), which is one’s perspective of the world when confronted by adversity or tension, and individuals with higher SOC are more resilient in practices that shape their health. For instance, an ageing employee is more likely to maintain the habit of performing PA if they are resilient against age-related declines in functional capacity. This idea is consistent with the import of the ATA, which asserts that people can maintain PA if they adapt past experiences to overcome age-related barriers (e.g., frailty) to social engagement and PA [1]. Life experiences shape SOC, enabling individuals to mobilise resources to cope with stressors and manage tension successfully [30].

Resilience can be shown successfully to maintain health with three qualities, namely *comprehensibleness*, *manageableness*, and *meaningfulness* [19, 30]. Comprehensibility is a cognitive quality, which means the ability to sense stimuli from the physical and social environment and detect stressors. We argue that WSH is a stimulus characterised by health-promoting practices such as workplace health education, the development of workplace policies to support health-seeking behaviours, and health advocacy by health champions at work. These tenets of the stimulus (i.e., WSH) can create an atmosphere of mentoring and learning, thereby enabling employees to develop and enhance their OHL and perform health-seeking behaviours that enhance their functioning and well-being. We define functioning as the physical and cognitive ability to independently perform essential daily tasks [11], including activities of daily living such as toileting and bathing. We adopt this definition because it best suits our measurement method and a work context where social, physical, and economic activities form the core of employees’ jobs.

**Fig. 1:**
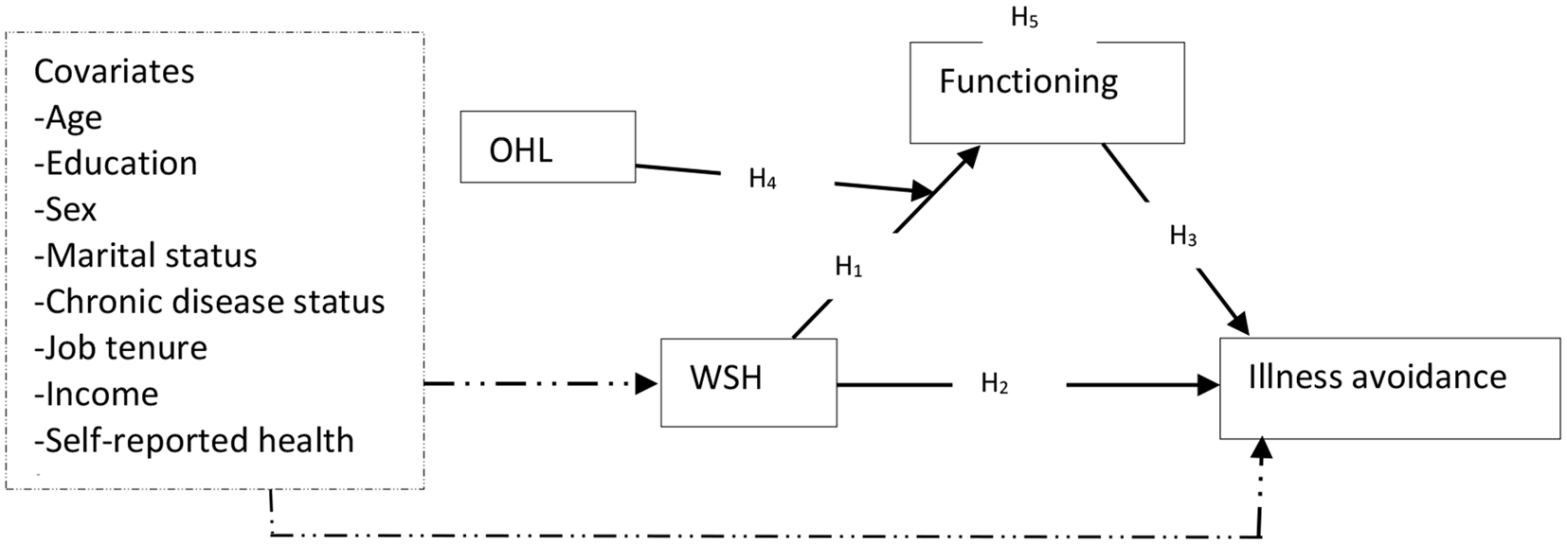
Conceptual model of the moderated mediation. *Note*: broken arrow represents confounding; CDS – chronic disease status, MS – marital status, SRH – self-reported health, WSH – workplace support for health; OHL – occupational health literacy. H1 – the effect of WSH on functioning; H2 – the effect of WSH on illness avoidance; H3 – the effect of functioning on illness avoidance; H4 – the moderating role of OHL in the effect of WSH on functioning; H5 – functioning mediates the effect of WSH on illness avoidance; H6 (not shown in the figure) – moderated mediation by OHL

WSH is designed to encourage behaviours [i.e., PA, social engagement, and healthy diet] that prevent the onset of disease [4, 6], and the ATA and CTA recognise functioning as both an antecedent and outcome of social participation and PA [1, 33]. Since WSH is a stimulus that can encourage PA, social engagement, healthy diet, and other health-seeking behaviours, it can serve as a process for the prevention of illness and improvement of functioning. These thoughts form the basis of the potential effect of WSH on functioning [hypothesis 1; H1] and illness avoidance [hypothesis 2; H2] in our moderated mediation model [see Fig. 1].

Manageability is a behavioural element of the theory that concerns the ability to draw on available resources to meet the demands of stressors [19, 30]. Stressors include any behavioural risks that make personal efforts to avoid ill health [e.g., too much sitting] abortive or an uphill struggle. As people age, their functional ability may decline [1, 34], but manageability can enable them to draw on their current functional ability to perform PA for a life without illness. With this trait of manageability, employees can make time out of their busy schedule and show resilience in regularly performing PA and other health-seeking behaviours. Manageability is analogous to the ability to adapt past experiences to overcome barriers to health-seeking behaviours as posited by the ATA and CTA [30]. These theories agree that the ability to perform daily tasks [which is implicit in functioning] protects health and longevity. Thus, employees with higher levels of functioning are more likely to maintain engagement with life in ways that prevent the onset and development of illness. This viewpoint underlies the potential effect of functioning on illness avoidance [hypothesis 3; H3] in our model.

Meaningfulness is a motivational dimension characterised by the individual’s motivation for coping with stimuli by investing energy into relevant protective behaviours [30, 35]. We reason that a source of motivation is OHL. People with a higher OHL may be better motivated to perform PA, eat healthy food, and avoid behaviours that threaten well-being since OHL is characterised by the ability to make good decisions about personal health [36]. Thus, meaningfulness provides the motivation to maintain functioning [through physical and cognitive abilities] and avoid illness since the salutogenic model argues that SOC can be enhanced through interventions that engage, raise awareness, and empower individuals for positive health behaviour. WSH is a measure of this programme and can be a driver of literacy and awareness about health risks and protective health-seeking behaviours.

Deductively, OHL may interact with WSH to influence functioning (hypothesis 4; H4), setting a foundation for the mediation (hypothesis 5, H5) and moderated mediation [hypothesis 6; H6] depicted in Fig. 1. This interaction is possible not only because WSH as a workplace stimulus can be associated with OHL, but also because other workplace factors outside WSH can positively influence OHL. An example is job design often not intended to be part of the organization’s WSH. Work experience and opportunities to learn from others within organizations, which can enhance OHL [6, 13], depend on job design. As illustrated in Fig. 1, the effects can be confounded by some personal factors. Education, for example, may influence employee or job rank, which would influence the level of WSH received. Men and women age in different ways and achieve different levels of PA [23, 24], which means that men and women may not have the same WSH. We, therefore, adjusted for the covariates shown in the model with a standard statistical technique.

**Fig. 2:**
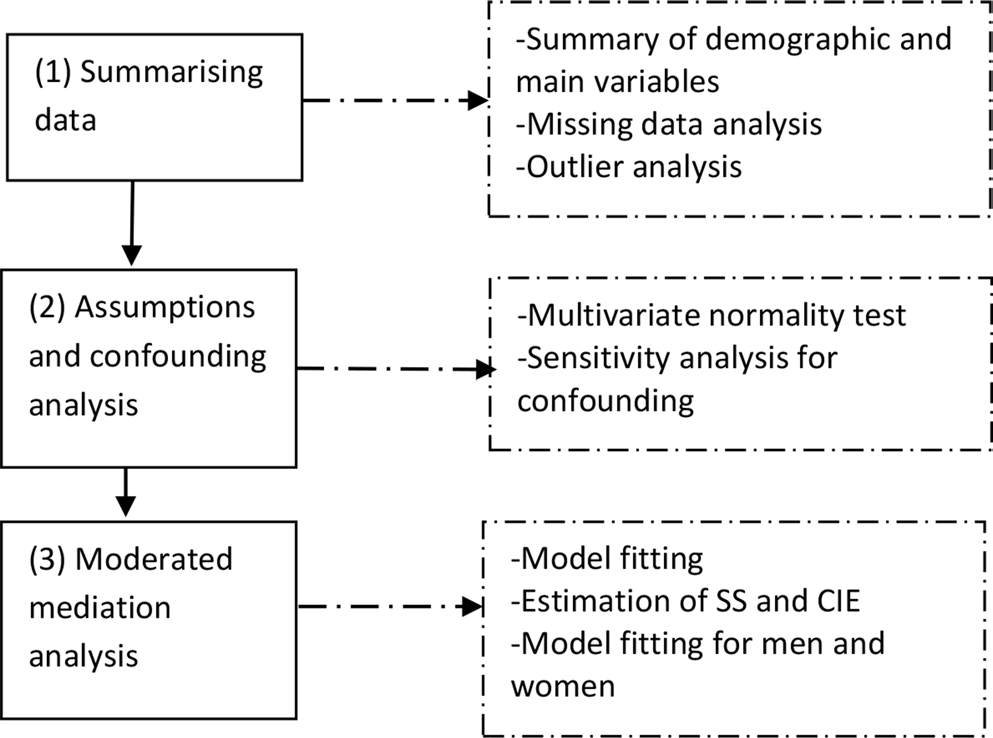
A flowchart of the statistical analysis method. *Note*: SS – simple slope; CIE – conditional indirect effect

## Methods

### Design

A cross-sectional design that follows the STROBE (i.e., Strengthening the Reporting of Observational Studies in Epidemiology) checklist was adopted. Figure 2 is a flowchart of the study design.

### Study setting, participants, and recruitment

The study setting was Accra, Ghana, and the participants were community-dwelling middle-aged and older adults aged 50 years or older. Multistage sampling was utilised to select the participants. We first classified the neighbourhoods of Accra into four cardinal blocks (i.e., north, south, west, and east) and randomly selected a representative number of neighbourhoods from each block. The participants were selected randomly from all selected neighbourhoods based on three selection criteria: (1) being aged 50 years or older; (2) being a permanent resident of Accra, and (3) availability and willingness to participate in the study. We calculated the minimum sample size necessary with the Daniel Soper’s sample size calculator for structural equation modelling [37, 38] based on standard statistics (i.e., moderate effect size = 0.3; power = 0.8, and α = 0.05). The sample size reached was 823, but we increased this number by 10% to allow for attrition. Thus, the minimum sample size of this study was 905.

### Variables, operationalization, and measures

WSH, the dependent variable, was measured with a 5-item scale with five descriptive anchors (i.e., 1 – strongly disagree, 2 – disagree, 3 – somewhat agree, 4 – agree, and 5 – strongly agree). This measure was adopted in whole from a previous study [9] and was developed based on our earlier definition of WSH. Some of its items are “Overall, my workplace supports me in living a healthier life” and “Most employees here have healthy habits”. It yielded satisfactory internal consistency in the form of Cronbach’s α ≥ 0.7 (overall α = 0.76; men’s α = 0.74; women’s α = 0.77), which is within the recommended cut-off point [9, 39]. Scores on this tool obtained by summing up its items range from 5 to 25, with larger scores indicating higher WSH.

Illness avoidance and functioning were measured with sub-scales from a previously validated successful ageing measure [11], associated with five descriptive anchors (i.e., 1 – strongly disagree, 2 – disagree, 3 – somewhat agree, 4 – agree, and 5 – strongly agree), and comprised 4 and 9 items respectively. Illness avoidance, the dependent variable, is a measure of overall health and the avoidance of medication as well as therapy. Some of its items are “I did not use medication or therapy” and “I was healthy enough to move around freely”. Functioning, the mediating variable, is a measure of cognitive functioning and how well one could perform physical and social tasks independently. Some of its items are “I had enough energy for daily life” and “When I tried to recall familiar names or words, it was not difficult for me to do so”. Illness avoidance (overall α = 0.72; men’s α = 0.76; women’s α = 0.81) and functioning [overall α = 0.84; men’s α = 0.87; women’s α = 0.80] produced Cronbach’s α ≥ 0.7. The ranges of scores on illness avoidance and functioning were 4–20 and 9–45 respectively, with larger scores indicating higher illness avoidance and functioning.

OHL, the moderating variable, was measured with a 12-item standard measure adopted in whole from a previous study [36]. The scale was associated with four descriptive anchors [i.e., 1 – strongly disagree, 2 – disagree, 3 – agree, and 4 – strongly agree] and produced satisfactory Cronbach’s α ≥ 0.7 (overall = 0.75; men's = 0.82; women's = 0.74). Its scores range from 12 to 48, with higher scores indicating higher OHL. Appendix 1 shows items used to measure WSH, illness avoidance, functioning, and OHL.

Eight potential covariates were measured following previous research [7, 34, 40, 41]. Chronic disease status was measured with a single question asking participants to report the number of chronic conditions they had, and the responses were coded into two groups (none – 1, and one of more – 2). Self-reported health was measured with a single question asking the participants to report whether their health was poor or good (poor – 1, and good – 2). Like chronic disease status and self-reported health, sex (men – 1, and women – 2), and marital status (not married – 1, and married – 2) were measured as categorical variables and coded into dummy-type variables. Job tenure, age, education, and income were measured as discrete variables. Job tenure was how long (in years) participants had worked in their current organization whereas age was a measure of chronological age. Education was measured as years of schooling whereas income was measured as the individual’s gross monthly earnings in Ghana cedis.

### Instrumentation

Data were collected with a self-reported questionnaire comprising three sections. The first section presented a statement of the study’s aim, importance, ethical considerations, and general survey completion instructions. The second section presented measures on WSH, OHL, functioning, and illness avoidance, whereas the final part captured questions on the covariates and personal factors. We avoided or minimised Common Methods Bias (CMB) at the survey design stage by following recommendations in the literature [34, 42, 43] to structure sections and questions in the questionnaire. In this vein, specific instructions for completing each scale and section in the right context were provided. The general instructions provided guided the participants to avoid errors in their completion of the survey. Standard scales with concise and unambiguous items or questions were used. In the second stage, Harman’s one-factor approach, a statistical procedure, was followed to investigate the absence of CMB in the data.

This technique required the use of an exploratory and confirmatory factor analyses to assess the factor structures of the scales used. With this technique, the absence of CMB in the data is confirmed if two or more factors are produced on each scale, or variances extracted are less than 40% [42, 43]. In the exploratory factor analysis, each scale yielded at least two factors, and each factor accounted for less than 40% of the total variance. WSH produced two factors (factor loadings of items ≥ 0.5; variance explained by factor 1 = 31.12, and variance explained by factor 2 = 23.09). OHL (number of factors extracted = 4), functioning (number of factors extracted = 3), and illness avoidance (number of factors extracted = 2) yielded similar results. Confirmatory factor analysis produced consistent results, signifying the absence of CMB in the data.

### Ethics and data collection

The study received ethical review and clearance from the ethics review board of the Africa Centre for Epidemiology (no. 005-10-2022-ACE) after the board reviewed the study protocol. All the participants provided written informed consent before participating in the study. We gathered data with three specially trained research assistants who administered questionnaires at designated centres. Some participants could not complete the questionnaire at the centres, so they were allowed to take the questionnaires home and return them over two weeks through a private courier hired by the researchers. Data were gathered over four weeks between July and August 2023. Out of 1501 questionnaires administered, 1015 were analysed, 465 were not returned by the participants, and 21 were discarded because at least 50% of their questions were not answered.

### Statistical analysis

We utilised SPSS 28 (IBM Inc., New York, USA) to summarise the data and perform exploratory data analysis, including the first sensitivity analyses for the ultimate covariates. Amos 28 was used to test the moderated mediation model. Data were summarised with descriptive statistics [i.e., frequency, and mean], enabling us to identify missing data. Marital status was the only variable with 1% missing data, but we performed the exploratory data analysis with the missing values as they were less than 10% (of the data on marital status) and were randomly distributed [34, 41]. We found no outliers in the data after using box plots to visualize the distribution of the data on all continuous variables. Previous studies [7, 34, 40] were then followed to perform the first sensitivity analysis for the ultimate covariates. We utilised this analysis to ensure that only measured covariates likely to confound the primary relationships were incorporated into the moderated mediation model. After following some standard steps (see Appendix 2), none of the measured covariates qualified as the ultimate covariate.

**Fig. 3:**
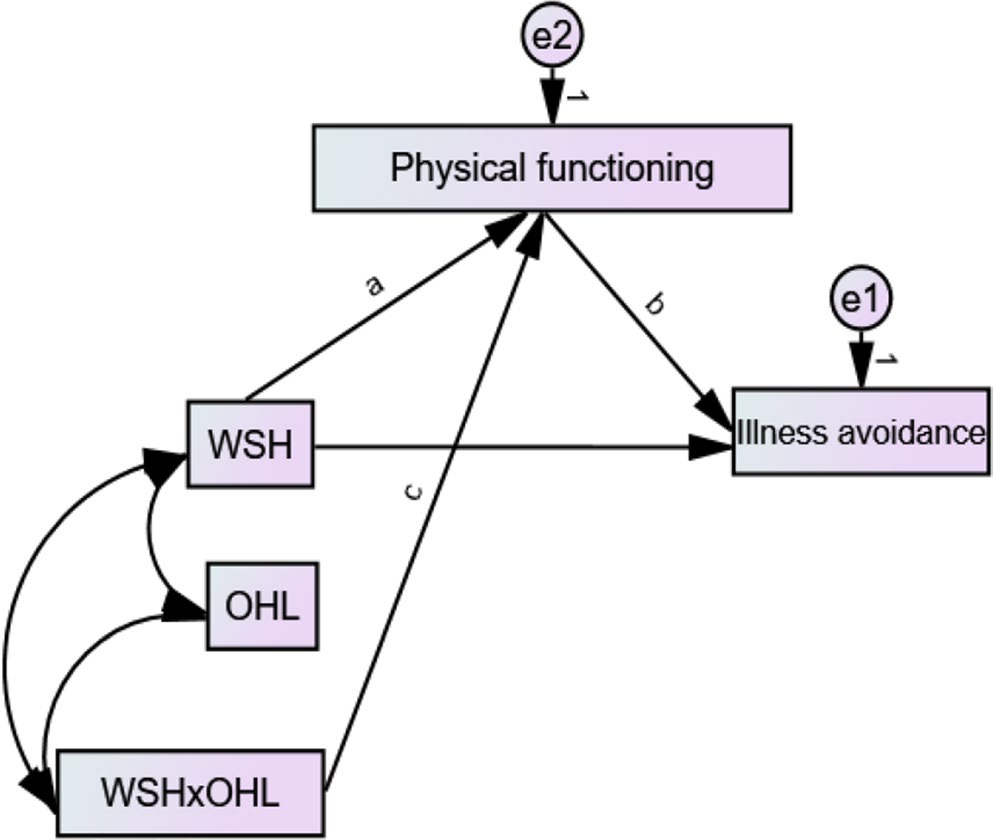
The statical moderated mediation model fitted. *Note*: WSH – workplace support for health; OHL – occupational health literacy; e1 and e2 are errors

Figure 3 shows the statistical moderated mediation model tested with Hayes’ Process Model [44, 45] through structural equation modelling. To create the interaction term (i.e., WSHxOHL), we centred the moderator (i.e., OHL) and multiplied it with WSH in harmony with Haye’s Process Model. The moderated mediation was fitted on the whole sample after computing the basic path coefficients (i.e., a, b, and c; see Fig. 3), Simple Slope (SS), Conditional Indirect Effect (CIE), and Index of Moderated Mediation (i.e., InModMed) using the “user-defined estimands” function in Amos 28. Appendix 3 shows the equations used to estimate the SS and CIE on the whole sample and sub-samples (i.e., men and women). The constant in each equation is the standard deviation of the moderator variable. The InModMed, SS, and CIE were estimated at different levels [i.e., low and high] of the moderator variable. The above parameters and their significance were based on 2000 biased corrected sampling iterations (bootstraps) with a 95% confidence interval.

In the second sensitivity analysis, the statistical model (see Fig. 3) was fitted for men and women, and the relevant parameters (i.e., direct effects, indirect effects, SS, CIE, and InModMed) were estimated for these samples. A minimum of *p* < 0.05 was used to detect the statistical significance of the effects. The moderation effect was visualized with figures depicting the effect of WSH on functioning at two levels (i.e., low, and high) of OHL. Multivariate normal distribution of the data was not achieved in fitting the models possibly owing to the relatively large sample used [46], but this violation of the assumption was corrected with our 2000 biased-corrected bootstraps [46].

**Table 1:** Summary characteristics on demographic and main variables (*n* = 1015)

Variable	M	SD	Range	*n*(%)
Sex				
Men				465(45.80%)
Women				550(54.20%)
Chronic disease status				
None				235(23.20%)
One or more				780(76.80%)
Marital status				
Not married				340(33.50%)
Married				665(65.50%)
Missing				10(1.00%)
Self-reported health				
Poor				260(25.60%)
Good				755(74.40)%
Job tenure (yrs)	10.60	4.71	3–30	
Income (C)	1643.09	1577.03	450–2500	
Age (yrs)	60.36	31.71	50–85	
Education (yrs)	19.32	5.42	9–26	
Workplace support for health	15.89	3.26	7–24	
Occupational health literacy	33.89	4.36	24–48	
Illness avoidance	13.80	2.15	8–20	
Functioning	17.14	2.49	10–23	

Note: M – mean; SD – standard deviation; n – frequency

**Table 2:** Direct and indirect effects of workplace support for health on illness avoidance

Outcome variable	Path	Predictor	Direct effects				Indirect effects	
			B	SE	Critical ratio	β	B	β
Men (*n* = 465)								
Functioning	<---	WSH	0.198	0.032	6.107	0.224***		
Illness avoidance	<---	WSH	0.106	0.031	3.421	0.147***	0.074***	0.103***
Illness avoidance	<---	Functioning	0.375	0.035	10.633	0.458***		
Functioning	<---	WSHxOHL	0.019	0.001	15.202	0.558***		

Women (*n* = 550)								
Functioning	<---	WSH	0.256	0.027	9.568	0.369***		
Illness avoidance	<---	WSH	0.200	0.023	8.838	0.321***	0.106***	0.170***
Illness avoidance	<---	Functioning	0.415	0.033	12.695	0.461***		
Functioning	<---	WSHxOHL	0.012	0.001	8.358	0.322***		

Whole sample (*n* = 1015)								
Functioning	<---	WSH	0.224	0.020	10.983	0.293***		
Illness avoidance	<---	WSH	0.166	0.019	8.981	0.253***	0.087***	0.132***
Illness avoidance	<---	Functioning	0.387	0.024	15.999	0.450***		
Functioning	<---	WSHxOHL	0.016	0.001	16.678	0.446***		

***p < 0.001; B – unstandardised effect; SE – standard error (of B); β – standardised effect; WSH – workplace support for health; OHL – occupational health literacy

## Results

In Table 1, about 46% [*n* = 465] of the participants were men, whereas the average age was about 60 years [Mean = 60.36; SD = 31.71]. The average WSH was about 16 [Mean = 15.89; SD = 3.26] whereas the average illness avoidance was 14 [Mean = 13.80; SD = 2.15]. WSH had a positive effect on illness avoidance [β = 0.29; *p* < 0.001] and functioning [β = 0.25; *p* < 0.001; see Table 2], which suggests that adult employees reporting higher WSH reported higher functioning and were more likely to avoid illness. Functioning had a positive effect on illness avoidance [β = 0.45; *p* < 0.001], which implies that adult employees with higher functioning were more likely to avoid illness. There was a significant indirect effect of WSH through functioning on illness avoidance [β = 0.13; *p* < 0.001]. The interaction term between WSH and OHL had a positive effect on illness avoidance [β = 0.45; *p* < 0.001].

**Fig. 4:**
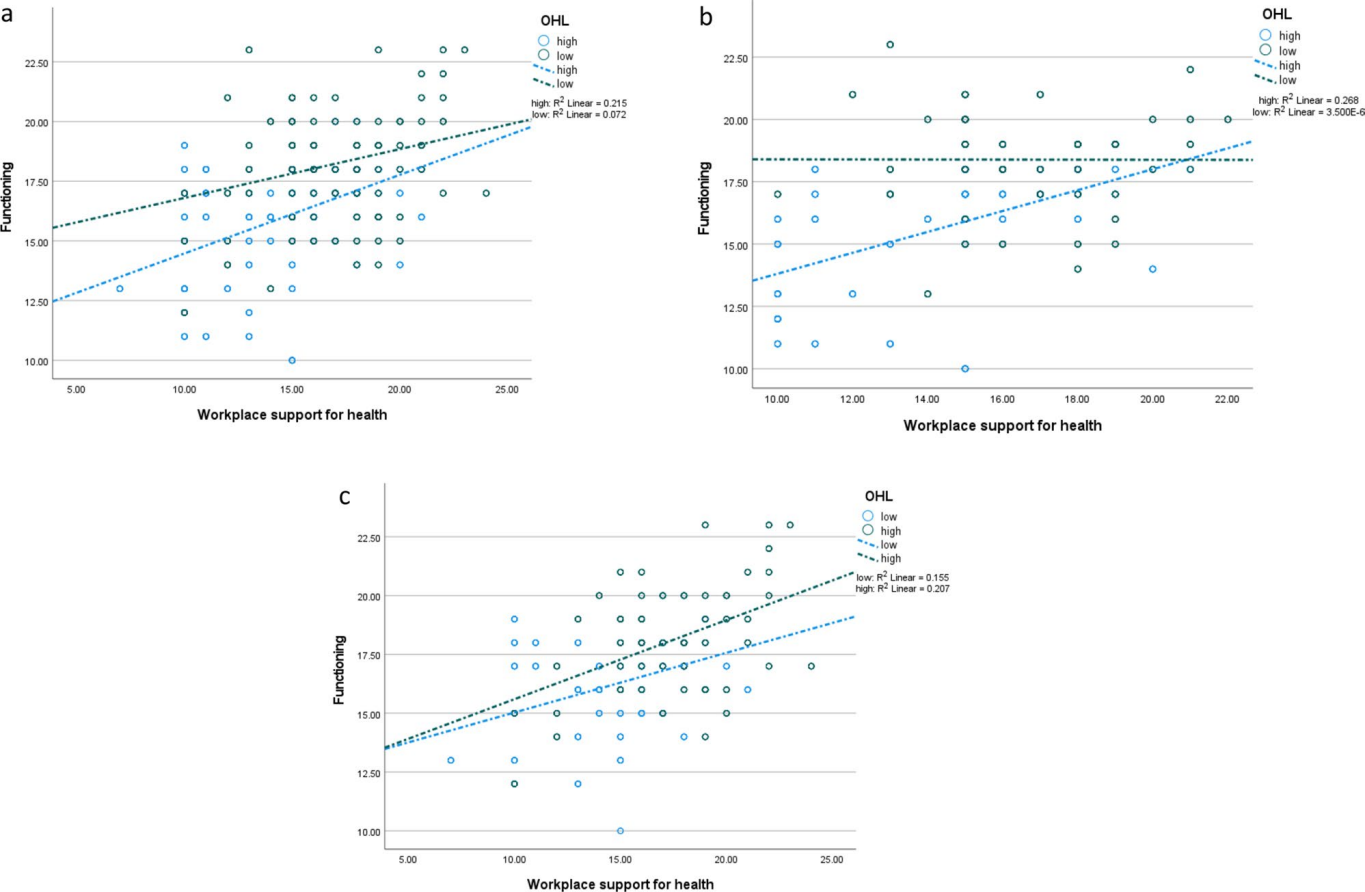
(**a**). Effect of workplace support for health on functioning at different levels (low = 508; high = 507; total = 1015) of occupational health literacy. *Note*: OHL – occupational health literacy. (**b**). Effect of workplace support for health on functioning at different levels (low = 233; high = 232; total = 365) of occupational health literacy among men. *Note*: OHL – occupational health literacy. (**c**). Effect of workplace support for health on functioning at different levels (low = 275; high = 275; total = 550) of occupational health literacy among women. *Note*: OHL – occupational health literacy

Among adult men, WSH had a positive effect on illness avoidance [β = 0.15; *p* < 0.001] and functioning [β = 0.22; *p* < 0.001] [see Table 2]. Functioning had a positive effect on illness avoidance [β = 0.46; *p* < 0.001], and the interaction between WSH and OHL had a positive effect on illness avoidance [β = 0.56; *p* < 0.001]. Similar effects were found for women, though the direct and indirect effects were stronger for women [compared with men] whereas the interaction effect was stronger for men. Figure 4a and b, and Fig. 4c depict the interaction effects on illness avoidance. In Fig. 4a, WSH had a stronger effect on functioning at higher OHL among men. Figure 4b and c show similar effects in women and the whole sample.

**Table 3:** Estimates of index of moderated mediation

Parameter	Men (*n* = 465)		Women (*n* = 550)		Whole sample (*n* = 1015)	
	B	95% CI	B	95% CI	B	95% CI
Estimates of simple slopes						
lowSS	0.103**	± 0.145	0.213***	± 0.120	0.168***	± 0.094
MedSS	0.216***	± 0.132	0.268***	± 0.111	0.240***	± 0.088
highSS	0.293***	± 0.127	0.299***	± 0.108	0.281***	± 0.086

Estimate of conditional indirect effects						
lowCIE	0.038**	± 0.055	0.088***	± 0.064	0.065***	± 0.041
medCIE	0.081***	± 0.060	0.111***	± 0.068	0.093***	± 0.046
highCIE	0.110***	± 0.070	0.124***	± 0.071	0.109***	± 0.050

Index of Moderated Mediation						
InModMed	0.007***	± 0.004	0.005***	± 0.003	0.006***	± 0.002

***p < 0.001; **p < 0.01; B – effect; SS – simple slope; CIE – conditional indirect effect; CI - confidence interval (of B); CI was based on 2000 biased-corrected sampling iterations

**Table 4:** Fit indexes of the three structured moderated mediated models

Index	Model		
	Men (*n* = 465)	Women (*n* = 550)	Whole sample (*n* = 1015)
Chi-square	94.920***	8.766	9.354
Goodness of Fit index	0.928	0.994	0.996
Tucker-Lewis Index	0.849	0.992	0.989
Root Mean Square Error of Approximation	0.257	0.059	0.046
Akaike Information Criterion	118.923	32.766	33.354

***p < 0.001

In Table 3, all estimates of simple slopes were significant at *p* < 0.001 in the overall sample and among men and women, which indicates that the effect of WSH on functioning was larger at higher levels of OHL. Estimates of conditional indirect effects were also significant in men and women and the whole sample, suggesting that the indirect effect of WSH on illness avoidance through functioning is stronger at higher OHL in the whole sample and for men and women. There was evidence of moderated mediation in the whole sample [parameter = 0.006; *p* < 0.001], and among men [parameter = 0.007; *p* < 0.001] and women [parameter = 0.005; *p* < 0.001]. This result means that the indirect effect of WSH through functioning on illness avoidance is moderated by OHL. Table 4 shows fit statistics from the structural models. Models based on women and the whole sample were a good fit.

## Discussion

This study investigated whether there is a moderated mediation by OHL in the relationship between WSH, functioning, and illness avoidance. The study found a positive effect of WSH on functioning, confirming the first hypothesis [H1] and implying that employees who had higher WSH reported higher levels of functioning. The *manageability* and *comprehensiveness* domain of the Salutogenic Model suggest that the individual can maintain life activities [e.g., social engagement, and PA] that enhance functioning after initially detecting and acting on the availability of resources [e.g., health information, workplace design, and safety kits] provided through WSH. The Activity Theory of Ageing and Continuity Theory of Ageing add that this ability can be maintained over the life course, implying that WSH would influence employee health across the lifespan if it sustains its salutary resources and support [e.g., training] for maintaining pro-health-seeking behaviours. Agreeing with our result is empirical research [4, 12, 47] confirming the positive effect of workplace health promotion programmes on PA-related aspects of functioning. Low physical functioning is analogous to frailty, which creates feelings of ill health [33, 48]. Our result implies from this viewpoint that WSH may be associated with lower incidences of self-reported ill-health and its consequential absences from work due to sickness.

This study found a positive effect of WSH on illness avoidance, which indicates that older employees in organizations with higher WSH were more likely to avoid illness. This result confirms the second hypothesis [H2] and is congruent with the salutogenic view that employees would understand and appreciate WSH as a workplace stimulus [i.e., *comprehensiveness*] and act on the stimulus in self-defence against disease [i.e., *manageability*]. We reason that health information sharing, physical workplace design, and the provision of occupational health support systems [e.g., policies, first aid, and safety measures] are salutary resources and stimulants in WSH that either encourage health-seeking behaviours [e.g., social engagement, PA, and healthy diet] or discourage behaviours that undermine individual health. Health champions at work [e.g., managers who are conscious about employee health] provide mentorship and share information in ways that encourage employees to avoid disease. They may invest in training and health promotion programmes that increase employees’ awareness of preventive behaviours such as exercise and healthy eating.

Empirical studies [4, 5, 13] have reported results similar to the link between WSH and illness avoidance, including the positive effect of WSH or workplace health promotion on health indicators such as the absence of burnout. Yet, our evidence is unique for linking a measure of workplace health promotion for the first time to illness avoidance, which is the desired outcome of salutogenic workplace programmes [10, 20].

There was a positive effect of functioning on illness avoidance, which confirms our third hypothesis [H3] and suggests that employees who reported higher functioning were more likely to avoid illness. This result is analogous to the positive association between functioning and indicators of well-being reported in the literature [49, 50] and is an extension of the impact of *manageability* on the individual in the sense that employees are more likely to report high illness avoidance if their functioning [a measure of physical ability implicit in *manageability*] is high enough. Our result, thus, corroborates our earlier argument that functioning is necessary for the avoidance of frailty, a measure of physiological limitations that implants in the individual feelings of ill health [33, 48]. So, individuals with higher functioning are more likely to feel healthy and avoid absences from work. This study is unique in linking functioning to illness avoidance, which is a more salutogenic view of the role of functioning in the productivity of employees.

The study confirmed the mediation of functioning in the effect of WSH on illness avoidance in support of the fifth hypothesis [H5]. Since WSH can influence illness avoidance directly without using its effect on functioning as a channel of influence, the above mediation is partial [51, 52]. A partial mediation in this vein reflects the value of WSH as a determinant of functioning and illness avoidance, both of which are necessary for multiple desired work outcomes such as individual job satisfaction and productivity. This value of WSH holds more meaning in situations where organizations prioritise cost optimisation through a reduction in expenditure relating to staff absences, early retirement, and health insurance payments.

Our data further supported the fourth and sixth hypotheses [H4 and H6], which are respectively about the moderation of the effect of WSH on functioning by OHL and the moderated mediation of OHL in our model. The moderation role indicates that the positive effect of WSH on functioning is stronger at different [higher] levels of OHL. The moderated mediation, on the other hand, means the indirect effect of WSH on illness avoidance through functioning is stronger at higher levels of OHL. Both effects signify that OHL complements the direct and indirect positive effects of WSH on illness avoidance. This evidence is supported by the SOC concept of the Salutogenic Model, which proposes that workplace health promotion provides an opportunity for learning and acquiring information for making good decisions about one’s health. Good decisions leading to healthy diet [53, 54], regular participation in PA [12, 13], and actions to avoid occupational health risks [15] are the outcomes of OHL, which is why OHL complements WSH within our moderated mediation model, possibly in the potency of the individual’s *manageability* or ability to savour salutary resources to perform health-seeking behaviours.

The above results from the consolidated data were confirmed in samples of men and women in our sensitivity analysis, though the effect sizes differed slightly between men and women. This outcome demonstrates the consistency of the evidence and the possibility of our evidence being supported in other samples or groups since similar previous group comparisons were used to ascertain the consistency of effects [25, 26]. Future studies may build upon our sensitivity analysis by fitting the model across other representative groups in workplaces. Future analyses can compare groups of income [i.e., low, moderate, and high earners], industry [i.e., service and manufacturing], and employment type [i.e., full-time, and part-time], given that these groups may not have the same opportunities at work.

The effect sizes of women were larger, which means WSH more strongly predicts illness avoidance directly and indirectly among women. This outcome could be because of women responding more positively to WSH programmes than men. A positive response to WSH programmes may include utilising health information provided to avoid health risks such as sedentary behaviour and unhealthy diet. Notably, the effects on men and women were positive, which signifies consistency between the two groups. These effects can be generalised to non-Ghanaian settings from the viewpoint of our theoretical framework. Thus, WSH is more likely to positively influence illness avoidance directly and indirectly at higher OHL in any context. Yet, WSH requires funding and logistics, so it may be more effective and regular in developed nations where organizations are better resourced to provide it. From this viewpoint, the effects reported in this study may be stronger in developed nations. The results suggest WSH and OHL play a role in illness avoidance, but WSH can only benefit individuals if they are employed. Employment in old age can foster social and financial autonomy as well as well-being. This study, thus, reinforces the need for individuals to maintain employability and employment as long as possible. National policies and interventions allowing ageing individuals to work in older age [e.g., after 60 years] are needed. An example of these policies is increasing the retirement age, given the increasing life expectancy in Ghana.

The results reinforce the importance of WSH and possibly other workplace health promotion programmes as a salutogenic way to enable ageing employees to avoid illness. Implicit in our salutogenic lens is the idea that workplace health promotion can help organizations achieve employee productivity by preventing disease. The ability of the organization to prevent the early onset of morbidities would save the cost of staff illnesses, early retirement, and absences from work. Since workplaces governed by a culture of disease prevention are, in theory, more productive than those built on a culture of disease treatment [6, 55], we encourage organizational-level investment in WSH to enable employees to age without illnesses. If organizations are cognisant of the long-term benefits of preventing the onset of diseases among their employees and ensuring that illnesses that can cause staff absences and early retirement are avoided, they can value and maintain this investment as a productivity strategy.

Salutogenic pathways to workplace health can make organizations more agile and sustainable since the avoidance of ill health at work enables organizations to retain productive staff for a longer period, avoid the cost of recruiting new staff to replace retired employees, and benefit from the unique work and social experiences of older employees. To realize these benefits, organizations need to incorporate workplace health promotion efforts targeting older or ageing employees into healthy ageing policies. This recommendation resonates with ongoing schools of thought on the important role of workplace ageing policies in organizations experiencing rapid employee ageing [56, 57]. Ideally, the above efforts should provide OHL as a personal resource for identifying workplace health risks and practising health-seeking behaviours, both of which are necessary precursors for functioning and illness avoidance. Whether improved within or outside WSH programmes, OHL would catalyse uptake of organization-led health initiatives by employees and improve employees’ health consciousness, engendering an organizational climate where good health over the life course is a shared output.

### Limitations and strengths

One of the limitations of this study was our use of the cross-sectional design, so we could not establish causation between the variables. As such, the effect sizes reached in this study ideally represent associations among the variables. Yet, this study provides estimates that may inform the design of experimental designs in the future. Effect sizes reported in this study may be used in sample size and power calculations in experimental research. The sample came from only Accra, so the results of this study may not be representative of older adults in Ghana or other cities of Ghana. Researchers and practitioners should be circumspect in using our results in non-Ghanaian or dissimilar contexts, and a replication of this study in other contexts where representative samples can be used is necessary. Future researchers can improve the scope of our evidence by assessing the stability or sensitivity of the results across contexts [e.g., urban versus rural settings] and other demographic factors [e.g., low, moderate, and high income]. The prospective or longitudinal design may be employed in future research to mitigate CMB and assess the stability of the relationships over time. Our measures were subjective or based on self-report; hence, this study was vulnerable to recall bias and social desirability bias. Even so, we tried to avoid or minimise response bias with standard procedures against CMB.

This study has several strengths. First, it is the first study to assess a link between WSH [a workplace promotion measure] to illness avoidance, thereby providing an evidence base for salutogenic workplace health promotion. Since workplace health promotion efforts are based on the principle of disease prevention [6], our study occupies a central place in the evidence to date and sets the basis for more future research linking measures of workplace health promotion [e.g., WSH] to illness avoidance. The Haye’s Process Model is superior to alternative approaches such as testing the model with hierarchical linear regression analysis, which would have increased the type 1 error of the analysis. The robustness of the analysis was maximised with our sensitivity analyses, which enabled us to avoid incorporating irrelevant variables into the moderated mediation model as covariates. Fitting the models between men and women also enabled us to assess the stability of the effects. Finally, our design followed theSTROBE checklist [58], which means the study met relevant quality markers. Appendix 4 shows items of the checklist met.

## Conclusion

Adult employees with higher WSH are more likely to report better functioning and avoid illness. Higher functioning is associated with a higher chance of illness avoidance, and the positive effect of WSH on functioning is stronger at higher OHL. The positive indirect effect of WSH on illness avoidance is stronger at higher OHL. The above results were consistent between men and women. The study concludes that WSH and OHL can individually and jointly be associated with higher functioning and a lower likelihood of illness. WSH and other programmes improving OHL are needed in organizations to support ageing employees to avoid illness. Future studies testing our models in other contexts, especially with representative samples and experimental designs, are needed.

## Electronic supplementary material

Below is the link to the electronic supplementary material.

Supplementary Material 1

Supplementary Material 2

Supplementary Material 3

Supplementary Material 4

Supplementary Material 5

Supplementary Material 6

Supplementary Material 4

## Data Availability

Data used for this study are available in the name DATA FILE_MEN and DATA FILE_WOMEN as an online supplementary material.

## Declarations

### Key to data file

OHL1-OHL12 – measure of occupational health literacy.

WSH1-WSH5 – measures of workplace support for health.

SA1-SA4 – measures of illness avoidance.

CDS – chronic disease status.

SRH – self-reported health.

#NULL! – missing data.
