# Supplementary material for: Association of workplace support for health with occupational health literacy and illness avoidance: moderated mediation by functioning through a salutogenic lens
Source: BMC Public Health. 2025 Aug 16;25:2816. doi: 10.1186/s12889-025-21831-3 (PMC12357482; doi:10.1186/s12889-025-21831-3)
Supplement: Supplementary file 3 — Supplementary Material 3 [file 12889_2025_21831_MOESM3_ESM.doc]

Appendix 3. Formulas used to compute simple slopes, conditional indirect effects, and index of moderated mediation

**Formula (whole data)**

lowSS=a+c*(-4.36468)

MedSS=a+c

highSS=a+c*(4.36468)

lowCIE=lowSS*b

medCIE=medSS*b

highCIE=highSS*b

InModMed=c*b

**Formular for men**

lowSS=a+c*(-5.09595)

MedSS=a+c

highSS=a+c*(5.09595)

lowCIE=lowSS*b

medCIE=medSS*b

highCIE=highSS*b

InModMed=c*b

**Formula for women**

lowSS=a+c*(-3.66359)

MedSS=a+c

highSS=a+c*(3.66359)

lowCIE=lowSS*b

medCIE=medSS*b

highCIE=highSS*b

InModMed=c*b

**Note**: The constants in the equations are the standard deviations of the moderator variable.
