# Supplementary material for: Association of workplace support for health with occupational health literacy and illness avoidance: moderated mediation by functioning through a salutogenic lens
Source: BMC Public Health. 2025 Aug 16;25:2816. doi: 10.1186/s12889-025-21831-3 (PMC12357482; doi:10.1186/s12889-025-21831-3)
Supplement: Supplementary file 5 — Supplementary Material 5 [file 12889_2025_21831_MOESM5_ESM.doc]

**Appendix 1**

**Appendix 1a. Measures of workplace support for health**

| # | Statement | 1 | 2 | 3 | 4 | 5 |
| --- | --- | --- | --- | --- | --- | --- |
| 1 | Overall, my workplace supports me in living a healthier life. |  |  |  |  |  |
| 2 | My supervisor supports me in living a healthier life. |  |  |  |  |  |
| 3 | Most employees here have healthy habits. |  |  |  |  |  |
| 4 | At my workplace, we have one or more leaders (e.g., CEOs or managers) who are wellness champions. |  |  |  |  |  |
| 5 | At my workplace, we have one or more employees who are wellness champions. |  |  |  |  |  |

**Note**: 1 – strongly disagree, 2 – disagree, 3 – somewhat agree, 4 – agree, and 5 – strongly agree

**Appendix 1b. Measures of functioning and illness avoidance**

| No | Item | 1 | 2 | 3 | 4 | 5 |
| --- | --- | --- | --- | --- | --- | --- |
|  | **Items for illness avoidance** |  |  |  |  |  |
| 1 | Bodily pain did not affect my performance at work or other essential tasks. |  |  |  |  |  |
| 2 | I did not use medication or therapy. |  |  |  |  |  |
| 3 | I was healthy enough to move around freely. |  |  |  |  |  |
| 4 | I had good health overall. |  |  |  |  |  |
|  | **Items for measuring functioning** |  |  |  |  |  |
| 1 | I had enough energy for daily life. |  |  |  |  |  |
| 2 | I have been sleeping well. |  |  |  |  |  |
| 3 | When I tried to recall familiar names or words, it was not difficult for me to do so. |  |  |  |  |  |
| 4 | I could perform two or more tasks simultaneously, for example, watching TV while discussing something else with another person. |  |  |  |  |  |
| 5 | My body and mind were strong enough to enable me to live independently without having others take care of me. |  |  |  |  |  |

**Note**: 1 – strongly disagree, 2 – disagree, 3 – somewhat agree, 4 – agree, and 5 – strongly agree

**Appendix 1c. Items for measuring occupational health literacy**

| SN | Potential/ability | 1 | 2 | 3 | 4 |
| --- | --- | --- | --- | --- | --- |
| 1 | Find safety and health information |  |  |  |  |
| 2 | Judge negative impact |  |  |  |  |
| 3 | Understand information |  |  |  |  |
| 4 | Implement proactive solutions |  |  |  |  |
| 5 | Change working conditions |  |  |  |  |
| 6 | Speak about health risks |  |  |  |  |
| 7 | Evaluate health promotion services |  |  |  |  |
| 8 | Find information about health risks |  |  |  |  |
| 9 | Inform about rules of conduct |  |  |  |  |
| 10 | Assume responsibility for health |  |  |  |  |
| 11 | Discussions with others |  |  |  |  |
| 12 | Active health promotion |  |  |  |  |

**Note**: 1 – strongly disagree, 2 – disagree, 3 – agree, and 4 – strongly agree
