# Supplementary material for: Association of workplace support for health with occupational health literacy and illness avoidance: moderated mediation by functioning through a salutogenic lens
Source: BMC Public Health. 2025 Aug 16;25:2816. doi: 10.1186/s12889-025-21831-3 (PMC12357482; doi:10.1186/s12889-025-21831-3)
Supplement: Supplementary file 7 — Supplementary Material 4 [file 12889_2025_21831_MOESM7_ESM.doc]

Appendix 2. Steps taken in the integrated sensitivity analysis

| Number | Action |
| --- | --- |
| Part 1 – Sensitivity analysis for the ultimate confounders | |
| 1 | Fit a simple linear regression model to assess the effect of the independent variable (IV) on dependent variable (DV) |
| 2 | Note the standardised regression weight (effect size) from step 1 |
| 3 | Fit a multiple linear regression model in which all measured covariates are treated as predictors of the IV |
| 4 | Identify from step 3 potential covariates that have a p-value ≥0.25 |
| 5 | Predictors from step 4 that produced a p≥0.25 should be removed from the analysis and the others kept for the next stage of the analysis |
| 6 | Adjust for each of the remaining covariates in the model fitted at step 1 |
| 7 | Compute the per cent (%) change between the standardised regression weight at step 1 and the new weight resulting from step 6 |
| 8 | All potential covariates that produce a change of 10% or more should be incorporated into the final analysis as the ultimate confounders |
| Part 2 – Comparison of the crude and adjusted models | |
| 1 | Fit the crude model that incorporates only the IV and DV* |
| 2 | Fit the adjusted model that incorporates the ultimate covariates into the crude model fitted in step 1 |
| 3 | Compare the standardised effect sizes (i.e., the effect of the IV on DV) of the crude and adjusted models and note the difference |
| Part 3 – Assessment of confounding influence between sub-groups and contexts | |
| 1 | Take the first two steps of part 2 for each of the sub-groups (e.g., smokers vs non-smokers) |
| 2 | Compare the standardised effect sizes (i.e., crude effects) between the sub-groups and note the differences* |
| 3 | Compare the standardised effect sizes (i.e., adjusted effects) between the sub-groups and note the differences** |
| 4 | Note changes in the crude and adjusted effect sizes between the sub-groups |
| 5 | Repeat actions 1 to 4 on any other sub-groups or contextual categories |

*the aim of the crude model is to fit a model estimating the effect of the independent variable on the dependent variable through a simple linear regression; **the aim of the adjusted model is to estimate the unique effect of the independent variable on the dependent variable; IV – independent variable; DV – dependent variable
